# Present and future of glass-ionomers and calcium-silicate cements as bioactive materials in dentistry: Biophotonics-based interfacial analyses in health and disease

**DOI:** 10.1016/j.dental.2013.08.202

**Published:** 2014-01

**Authors:** Timothy F. Watson, Amre R. Atmeh, Shara Sajini, Richard J. Cook, Frederic Festy

**Affiliations:** King's College London Dental Institute, Biomaterials, Biomimetics & Biophotonics (B^3^), Floor 17, Guy's Tower Wing, Guy's Hospital, London Bridge SE1 9RT, United Kingdom

**Keywords:** Bioactivity, Calcium silicate, Glass ionomer, Cements, Caries, Remineralization, Biophotonic imaging

## Abstract

**Objective:**

Since their introduction, calcium silicate cements have primarily found use as endodontic sealers, due to long setting times. While similar in chemistry, recent variations such as constituent proportions, purities and manufacturing processes mandate a critical understanding of service behavior differences of the new coronal restorative material variants. Of particular relevance to minimally invasive philosophies is the potential for ion supply, from initial hydration to mature set in dental cements. They may be capable of supporting repair and remineralization of dentin left after decay and cavity preparation, following the concepts of ion exchange from glass ionomers.

**Methods:**

This paper reviews the underlying chemistry and interactions of glass ionomer and calcium silicate cements, with dental tissues, concentrating on dentin–restoration interface reactions. We additionally demonstrate a new optical technique, based around high resolution deep tissue, two-photon fluorescence and lifetime imaging, which allows monitoring of undisturbed cement–dentin interface samples behavior over time.

**Results:**

The local bioactivity of the calcium-silicate based materials has been shown to produce mineralization within the subjacent dentin substrate, extending deep within the tissues. This suggests that the local ion-rich alkaline environment may be more favorable to mineral repair and re-construction, compared with the acidic environs of comparable glass ionomer based materials.

**Significance:**

The advantages of this potential re-mineralization phenomenon for minimally invasive management of carious dentin are self-evident. There is a clear need to improve the bioactivity of restorative dental materials and these calcium silicate cement systems offer exciting possibilities in realizing this goal.

## 1 Introduction

The interaction between restorative dental materials and tooth tissue encompasses multiple aspects of dental anatomy and materials science. Until relatively recently, many adhesive dental restorative materials were thought to have a passive hard tissue interaction based on simple infiltration with the enamel or dentin upon which they were placed. However, there is increasing interest in mapping the interactions between materials and tooth tissue, where the former has a more aggressive interaction with the latter, while promoting ‘bioactivity’; it can be argued that materials such as glass ionomer cements have had such interactions for over thirty years, but only recently have mechanisms of adhesion and adaptation been elucidated indicating the potential for chemical interactions with both resin [1] and water-based cement-like materials [2]. Bioactivity can be defined as ‘materials that elicit a specific biological response at the interface between tissues and the material, which results in the formation of a bond’. In this paper we will concentrate on two classes of water-based cement-type restorative materials, glass ionomer and calcium-silicate based cements, and examine their interactions with the hard tissues in health and disease, in particular examining their potential for bio-mineralization in dentin. These materials are promoted as dentin replacements, mimicking many of the physical properties of this composite biological material, but they do not, as yet, have the wear resistance and mechanical properties to make them suitable as long-term enamel replacements.

## 2 Glass ionomer cements

Glass ionomer cements (GICs) were first introduced to dentistry in 1975 [3] and since then they have been used in a wide range of clinical applications. Conventional GICs are dispensed in a powder form supplied with its own liquid. The powder is formed of fluoro-aluminosilicate glass, while the liquid is an aqueous solution of a polyalkenoic acid, such as polyacrylic acid, although in later formulations, the acid may be added to the powder in a dried polymer form [3]. Strontium has been added in some commercial GICs, such as GC Fuji IXGP (GC Corporation, Tokyo, Japan), to substitute calcium due to its radiopaque properties [4]. This substitution does not have any effect on the setting products [5] or cement's remineralizing capability [6].

Upon mixing, an acid–base reaction takes place between the polyalkenoic acid and the ion-leachable glass particles [7], which occurs in two phases. The first phase is a dissolution phase, in which the acid attacks the surface of the glass particles to release ions such as aluminum, fluoride, and calcium or strontium. Following ion release, the polyacid molecules become ionized and adopt a more linear form. This renders the polyacid's carboxylic groups more accessible for the ions and facilitates their cross linking in the later stage of gelation [7]. Eventually, the set cement will be formed of a composite of un-reacted glass particle cores, encapsulated by a siliceous gel and embedded in a polyacid–salt matrix which binds the components together [8], [7], [2]. Within the polyacid–salt matrix of set GICs, water is distributed in two forms; loose water, which can be removed through desiccation, and bound water which is chemically locked into the matrix [3]. Water plays an essential role during the maturation of the cement as well as the diffusion of ions [4].

Further material developments have included a newly named material: ‘Glass Carbomer^®^’ (GCP Dental, Mijlweg, Netherlands) which is claimed to contain nano glass particles, hydroxyapatite/fluorapatite (HAp/FAp) nano particles and liquid silica. The nanocrystals of calcium fluorapatite (FAp) may act as nuclei for the remineralization process and initiate the formation of FAp mineral as well as nanocrystals of hydroxyapatite (HAp). The glass has a much finer particle size compared to conventional GICs, giving properties that are thought to aid its dissolution and ultimate conversion to FAp and HAp. However, using “magic angle spinning” nuclear magnetic resonance spectroscopy, Zainuddin et al. [9] have shown that the HAp in the powder is consumed during the cement formation process in this material and so may in fact have reduced availability for bio-mineralization.

When a freshly mixed GIC is placed on wet dentin, an interaction between the two materials takes place, in the form of an ion exchange [10], [11]. Aluminum, fluoride, and calcium or strontium leach out of the cement as the glass is being dissolved by the polyacid, while calcium and phosphate ions also move out of the underlying dentin as a result of the self-etching effect of the setting cement on mineralized dentin [10], [12]. This ion exchange process creates an intermediate layer composed of ions derived from both substrates [2], [12], [11]. The release of fluoride and calcium/strontium ions has provided GICs with the potential for remineralization of carious tissues [13], [6], where ion exchange could replenish the demineralized tissues’ ions, thus tipping the balance in favor of apatite (re-)formation.

## 3 Calcium-silicate based cements

Calcium-silicate based cements were first introduced to dentistry in 1993 when Torabinejad developed a formula based on ordinary Portland cement (OPC) to produce the mineral trioxide aggregate, or the gray MTA [14]. This material was principally composed of tri-calcium silicate, di-calcium silicate, tri-calcium aluminate, and tetra-calcium aluminoferrite, in addition to calcium sulphate and bismuth oxide, added as a radiopaquer for clinical applications [15], [16], [17]. In 2002, a white MTA version was developed, which was identical to the gray form but lacked the tetra-calcium aluminoferrite [16], [18] and had reduced aluminate levels [19]. Since their introduction, MTAs have been principally used for endodontic applications such as repairing perforated roots, apexification, or pulp capping [20], [21], [22] due to their relatively long working and setting times.

In 2011, Biodentine™, a quick-setting calcium-silicate based dental cement, was introduced by Septodont (Saint Maur des Fosses – France). Henceforth this commercial name will be used for representation and brevity. Biodentine™ was developed as a dentin replacement material, a novel clinical application of this family of materials, intending it to function as a coronal restoration. The relatively short setting time (around 12 min), can enable the use of this cement for restorative procedures; impossible with MTAs that achieve an initial setting 3–4 h [23]. Biodentine™ is principally composed of a highly purified tri-calcium silicate powder that is prepared synthetically in the lab de novo, rather than derived from a clinker product of cement manufacture. Additionally, Biodentine™ contains di-calcium silicate, calcium carbonate and zirconium dioxide as a radiopacifer. The di-calcium and tri-calcium silicate phases form around 70% of the weight of Biodentine's de-hydrated powder, which is close to that of white MTA and white Portland cement [24], [25]. Unlike MTA, Biodentine does not contain calcium sulphate, aluminate, or alumino-ferrate. The powder is dispensed in a two part capsule to which is added an aliquot of hydration liquid, composed of water, calcium chloride, and a water reducing agent.

Despite similar constituents, there is significant variation in calcium-silicate dental cement manufacturing processes. This affects the purity of their constituents and hydration products, as well as their behavior [26]. Studies have shown that cements such as ProRoot MTA (Dentsply, Tulsa Dental Products, Tulsa, OK) and MTA Angelus (Angelus Soluções Odontológicas, Londrina, Brazil) share almost the same composition as white OPC, except for the addition of bismuth oxide to the MTAs, for radiopacity [15], [27], [22]. However, the MTAs also include impurities and contaminating heavy metals such as chromium, arsenic, and lead [28]. This suggests their manufacture is similar to OPCs but less segregated and refined as the particle sizes also vary more widely [21]. On the other hand, other calcium silicate based dental cements, such as Biodentine™ and MTA-bio (Angelus Indústria de Produtos Odontológicos Ltda, Londrina, Brazil) have been produced under more stringent production conditions from raw materials, in an attempt to avoid any potential contamination of the basic constituents, and to avoid the incorporation of aluminum oxide [26].

Similar to OPC, calcium-silicate based dental cements set through a hydration reaction [19], [24], [26]. Although the chemical reactions taking place during the hydration are more complex, the conversion of the anhydrous phases into corresponding hydrates can be simplified as follows:

2Ca_3_SiO_5_ + 7H_2_O → 3CaO·2SiO_2_·4H_2_O + 3Ca(OH)_2_ + energy

C_3_S + water → CSH + CH

2Ca_2_SiO_4_ + 5H_2_O → 3CaO·2SiO_2_·4H_2_O + Ca(OH)_2_ + energy

C_2_S + water → CSH + CH

This setting reaction is a dissolution–precipitation process that involves a gradual dissolution of the un-hydrated calcium silicate phases (C_3_S and C_2_S) and formation of hydration products, mainly calcium silicate hydrate (CSH) and calcium hydroxide (CH). The CSH is a generic name for any amorphous calcium silicate hydrates which includes the particular type of CSH that results from the hydration of tri- and di-calcium silicate phases [29]. The CSH precipitates as a colloidal material and grows on the surface of un-hydrated calcium silicate granules, forming a matrix that binds the other components together, gradually replacing the original granules. Meanwhile, calcium hydroxide is distributed throughout water filled spaces present between the hydrating cement's components [29], [30]. Compared with OPC, the hydration of MTA is affected by the bismuth oxide, which forms 20% of its weight and acts as a radiopacifier [19], [24]. Hydrated MTA and tri-calcium silicate cements were found to release more calcium ions than hydrated OPC [19], [26].

For Biodentine™, the setting reaction is expected to be similar to the hydration of pure tri-calcium silicate cement [26] and is therefore expected to produce the same hydration products as WMTA and OPC, except for the absence of alumina and gypsum hydration products. The zirconium oxide present in the Biodentine™ powder as a radiopacifier also acts as an inert filler and is not involved in the setting reaction [26], unlike the bismuth oxide present in MTA [19]. The hydration reaction starts with the fast dissolution of the tri-calcium silicate particles, which therefore can explain the fast setting of Biodentine™ [29], in addition to the presence of calcium chloride in the liquid, which is known to speed up the hydration reaction [30], and the absence of calcium sulphate that acts as a retarder.

Despite similar contents, the manufacturing variations and absolute constituent ratios influence clinical behavior within the materials group. Therefore, it is essential to study and characterize these cements individually, in order to understand their nature and clinical behavior. Such studies have been limited for Biodentine™ [31], [32], [33], [34]. Therefore, further characterization of Biodentine™ and its setting reaction is required. However, a number of studies have been conducted on the interaction of Biodentine™ with dental tissues [35], [36], [37], [38] and pulpal tissues [39], [40], [41].

**Fig. 1:**
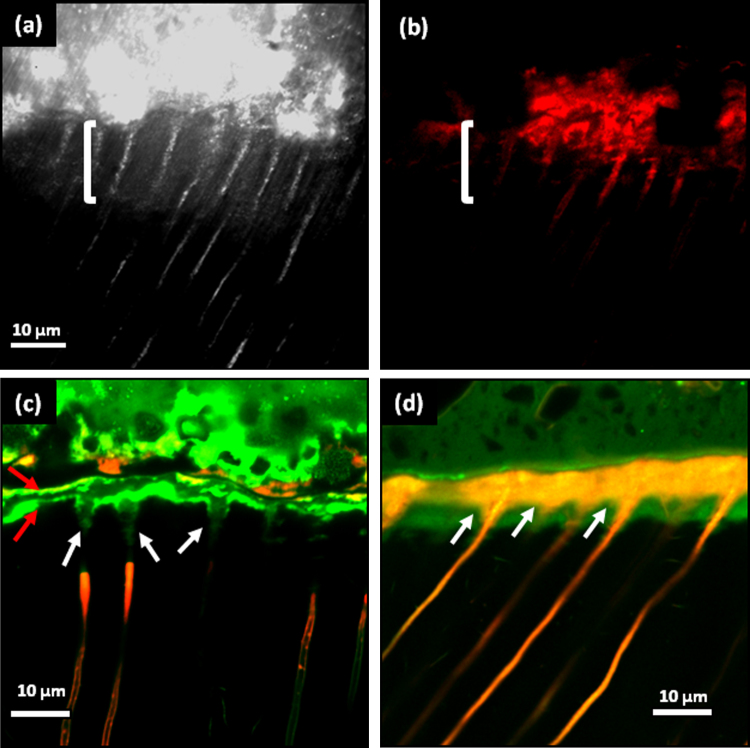
(a) Reflection mode confocal image of the dentin–Biodentine™ interface showing the highly reflective band (bracket). The micro-permeability of the Rhodamine-B dye solution was reduced which was demonstrated by the fluorescence mode image of the same area (b). (c) The mineral infiltration zone (MIZ) is characteristic for the interfacial dentin underneath the Biodentine™ cement: tag-like structures forming within the dentinal tubules (tip of arrow) have interrupted the dye solution permeating from the pulp chamber (base of arrows). (d) The ion exchange layer in the dentin–GIC interface with the scalloped appearance in the inter-tubular dentin (arrows) as a result of the acidic nature of the setting cement demineralizing the intra-tubular dentin.

The interfacial properties of Biodentine™ and a glass-ionomer cement (GIC Fuji IXGP) with dentin have been studied using confocal laser scanning microscopy (CLSM), scanning electron microscopy (SEM), micro-Raman spectroscopy, and two-photon auto-fluorescence and second harmonic-generation (SHG) imaging by Atmeh et al. [38]. Their results indicated the formation of tag-like structures alongside an interfacial layer called the “mineral infiltration zone”, where the alkaline caustic effect of the calcium silicate cement's hydration products degrades the collagenous component of the interfacial dentin. This degradation leads to the formation of a porous structure that facilitates the permeation of high concentrations of Ca^2+^, OH^−^, and CO_3_^2−^ ions, leading to increased mineralization in this region. Comparison of the dentin–restorative interfaces shows that there is a dentin-mineral infiltration with the Biodentine™, whereas polyacrylic and tartaric acids and their salts lead to the diffuse penetration of the GIC; consequently a new type of interfacial interaction, “the mineral infiltration zone”, is suggested for these calcium-silicate-based cements (Fig. 1a–d).

## 4 Dentin remineralization

For remineralization of dentin, different approaches have been applied, which can be classified as classical and non-classical [42], or top-down and bottom-up approaches [43]. In the classical (top-down) approach [44], [45], [46], [47], [48], dentin remineralization is based on the epitaxial growth of residual crystallites, which act as nucleation sites for the calcium phosphate minerals to precipitate when dentin is stored in a solution rich with calcium and phosphate ions [43]. However, recent studies have indicated that such an approach would result in an incomplete and non-functional remineralization of dentin [49], [50], [51], [52]. The classical remineralization approach results in extra-fibrillar remineralization of the collagen matrix of dentin, without the mineralization of the collagen's intra-fibrillar compartments [50]. This is due to the lack of control on orientation and size of the apatite crystals formed during this process. The non-classical approach was suggested as an alternative in vitro remineralization system, which attempted to achieve a hierarchical biomimetic remineralization of the organic matrix of dentin [49]. This approach involves the use of synthetic substitutes for certain dentin matrix proteins that play an essential role during the bio-mineralization process. Two types of analogs were suggested: the first is a sequestration analog, which requires polyanionic molecules such as polyacrylic acid to allow the formation and stabilization of amorphous calcium phosphate (ACP) [49], [50], [53]. These nano-aggregates of ACP are thought to form flowable nano-precursors which can infiltrate the water filled gap zones in dentinal collagen fibrils, where they precipitate as polyelectrolyte-stabilized apatite nano-crystals [49]. This precipitation is guided by the second analog, which is a dentin matrix phosphoprotein substitute [54]. This analog is usually a polyphosphate molecule, such as sodium metaphosphate, which acts as an apatite template, encouraging crystalline alignment in the gap zones [53], leading to a hierarchical dentin remineralization [50].

In the biomimetic bottom-up remineralization approach, calcium silicate based cements, such as MTA, were used as the calcium source [49], [54], [53]. Upon hydration, these cements release calcium in the form of calcium hydroxide over a long duration [26]. In addition to calcium provision, these cements release silicon ions into underlying dentin [35]. Silica was found to be a stronger inducer of dentin matrix remineralization compared to fluoride [48]. Furthermore, calcium silicate cements have the advantage of high alkalinity, which favors apatite formation [55] and matrix phosphorylation [52], thereby providing a potential caustic proteolytic environment which could enhance dentin remineralization [46].

The non-classical approach is thought to be advantageous over the classical, as the former provides continuous replacement of water, which occupies the intra-fibrillar compartments, by apatite crystals. The non-classical approach also provides a self-assembly system that does not require the presence of nucleation sites [43]. However, the idea of using matrix protein analogs could be challenging clinically and difficult to apply. Recent developments have attempted to take this concept a further step into clinical application [53]. Therefore, the classical approach remains a closer and more applicable approach to the clinical situation.

## 5 Imaging dentin mineralization

Many different techniques have been used to evaluate dentin mineralization. Scanning and transmission electron microscopy (SEM and TEM) [56], [49], Fourier transform infrared spectroscopy (FTIR) [57], Raman spectroscopy [58], X-ray diffraction (XRD) [48], energy dispersive X-ray spectroscopy (EDX) [52], microradiography [45], [47], micro-CT scanning [59], and nano-indentation [51]. However, they do not enable high-resolution observation of this process within the organic matrix of dentin. The capability of these techniques in detecting mineralization at high resolution is limited to surface characterization, whether morphological, chemical, or mechanical. Hence, they do not enable the observation of the mineralization process and its morphological features deeply within the structure of the remineralizing organic matrix and may not allow a return to the same sample position over a period of time. For this purpose, combining a microscopic technique with the capability of deep tissue imaging, such as two-photon fluorescence microscopy, with a selective mineral labeling fluorophore, such as Tetracycline, could provide a useful method [60].

Tetracyclines are polycyclic naphthacene carboxamides, composed of four carboxylic rings, which facilitate binding of the molecule to surface available calcium ions of the mineralized tissue. This binding process, helpfully enhances the fluorescence of Tetracycline after binding to calcium in these tissues [61]. Therefore, this labeling agent has been widely used for the study of mineralization process in bone [62], [63], and teeth [64], [65]: it is normally excited in the deep blue (400 nm) excitation range.

Two-photon excitation fluorescence microscopy is an advanced imaging technique that enables high-resolution observation of deep tissues due to the reduced scattering of high-intensity pulsed infrared laser light compared with conventional confocal microscopy [66], [67]. Our home-built two-photon lifetime fluorescence microscope uses a femtosecond Ti-Sapphire laser to excite the sample and a time-correlated single-photon counting card to record the lifetime of the emitted fluorescence, providing extra intensity-independent imaging contrast. Measurement of the subtle fluorescence lifetime variations across a dentin–restoration interface allows the sensitive detection of newly-formed Tetracycline-bound minerals.

**Fig. 2:**
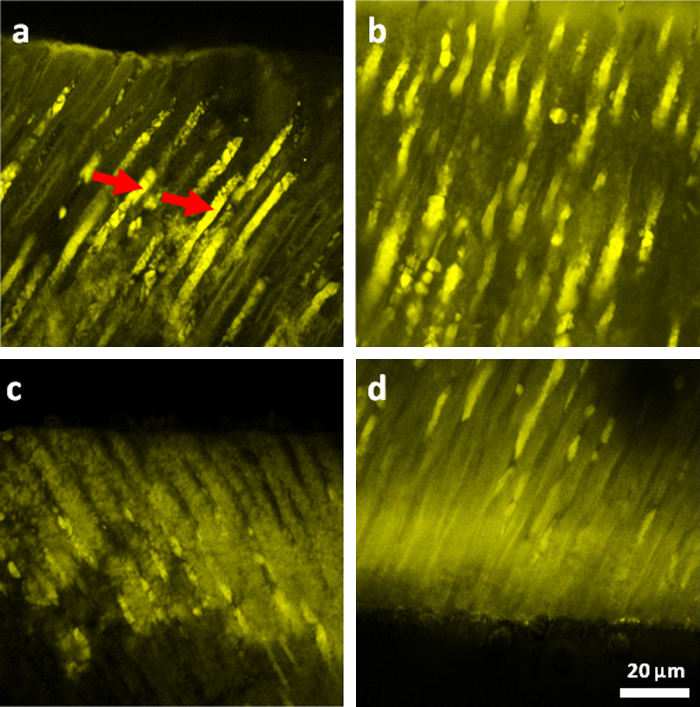
Mineralization detection with Tetracycline labeling and two-photon fluorescence imaging. (a) Intra-tubular mineralization in the form of highly fluorescent structures within the dentinal tubules (arrows). (b) Intra-tubular mineralization with globular appearance. A highly fluorescent band is present within the dentin just underneath the cement (c), as well as on the opposing dentin surface of the demineralized disk (d).

In a study reported by Atmeh et al. [60] 1.5 mm thick demineralized dentin disks were apposed to set calcium silicate dental cement: Biodentine™. The samples were stored in PBS solution with Tetracycline and kept in an incubator at 37 °C. Disks of demineralized dentin were kept separately in the same solution but without the cement to be used as control samples. After 6 weeks, one half of each sample was examined using a two-photon fluorescence microscope, illuminating at 800 nm and with a 40× objective (Fig. 2). A highly fluorescent Tetracycline band was detectable beneath both the apposed and under-surface of the disks (Fig. 2c and d). Small globular structures were also noticed in the tubular walls adjacent to highly fluorescent substances within the dentinal tubules (Fig. 2a and b).

With the enhanced understanding of the dental caries process and improvements in both dental materials and diagnostic devices, more interest has been directed toward minimally invasive approaches for the treatment of dental caries. Such approaches eventually aim to minimize the excavation of dental tissues and instead, to encourage their recovery and repair. Dentin caries results from a bacteriogenic acid attack followed by enzymatic destruction of the organic matrix. These lesions can be classified into caries-infected and -affected dentin [68] based on the extent and reversibility of the damage induced. In the caries-infected dentin, the organic matrix is irreversibly damaged, while the deeper caries affected dentin is hypomineralized with sound organic matrix, which could be repaired and remineralized. The slow progression of caries allows a reparative intervention, which can restore the mineralized architecture after excavating the infected layer.

## 6 Carious dentin remineralization models

Carious dentin remineralization has been studied extensively using different in vitro models. In these models, the dentin caries process has been simulated by partial demineralization of sound dentin using pH cycling [59] or short duration application of phosphoric acids [44] or ethylenediaminotetraacetic acid (EDTA) [47] and in some studies total demineralization [45] and [46]. These methods were actually aiming to simulate the caries affected “hypomineralized” dentin, which has the potential to be remineralized. However, these models all show shortcomings in that they do not model the effect of the pulpal response to the carious lesion and the tissue fluid dynamics/re-mineralization phenomena within the dentin tubules. The challenge of using real carious dentin as a substrate is its inherent variability, so methods of characterizing the extent of caries removal and the position of the tooth restoration interface within the treated lesion would be beneficial for introducing some degree of consistency to a variable substrate. The use of confocal endoscopic fluorescence detection methods can aid in this depth determination and discrimination [69], while other optical techniques such as Raman spectroscopy and fluorescence lifetime imaging may also help to characterize the dentin caries substrate [70], [71].

## 7 Biophotonics-based characterization of the caries affected dentin–cement restoration interface

**Fig. 3:**
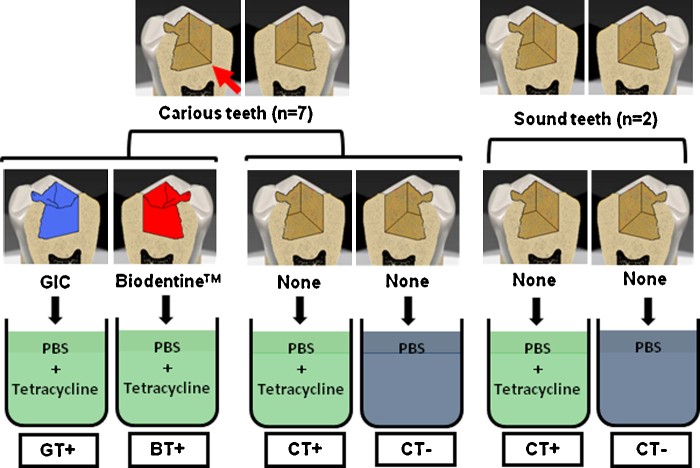
Sample preparation. Deep carious lesions in extracted human teeth were accessed; a right angle between the mesial wall and pulpal floor of the cavity (arrow) was created using a cylindrical bur. Caries infected dentin was excavated and each tooth was sectioned through the cavity into 2 halves, each was restored with a different restorative material; Biodentine™ (right), or GIC (left), four teeth were left without restorations. Samples were stored for 8 weeks in phosphate buffered saline (PBS) with or without Tetracycline, (T+) or (T−) respectively. C: carious dentin; S: sound dentin.

Recent work in our laboratory has investigated the capability of Biodentine™ and GICs to induce re-mineralization in caries affected dentin. In order to give a reasonably reliable indicator of caries excavation endpoint [69], caries infected dentin was excavated chemo-mechanically using Carisolv^®^ gel (MediTeam Dental, Göteborg, Sweden) in seven carious extracted teeth (Fig. 3). Excavation was performed after preparing occlusal cavities to access the carious lesions. On one of the proximal walls of the cavity (Fig. 3), a diamond cylindrical bur was used to prepare a flat proximal wall to the cavity to create a right angle with its floor; this angle was used later as a fiducial reference for the imaging. Two additional sound teeth were used as controls, in which occlusal cavities were prepared and left without restoration. For each tooth, the root was cut and the crown was sectioned vertically through the middle of the cavity into two halves using a water-cooled wafering blade. Sectioned samples were subsequently polished using 600, 800, and 1200-grit carborundum papers and cleaned in an ultrasonic bath with deionized water for 10 min.

The halves of five caries-excavated sectioned cavities were filled with two different restorative materials; one with Biodentine™ and the other glass ionomer cement Fuji IXGP (GC Corporation, Tokyo, Japan) (Fig. 3). Before applying the cements, each sectioned tooth was mounted using a specially designed small vice, with which the sectioned surface of the tooth was tightened against a rigid plastic matrix to prevent any leakage of the cement. Cements were applied directly after mixing as per manufacturer's instructions using an amalgam carrier for the Biodentine™ and adapted with a plastic instrument. Samples were stored for 1 hour in an incubator at 37 °C temperature and 100% humidity. For aging, samples were stored in a 0.015% Tetracycline solution (87128 Sigma–Aldrich, Dorset, UK) and phosphate buffered saline (PBS) (Oxoid Limited, Hampshire, UK) at 37 °C for 8 weeks. Solutions were replaced every 2 days. Two carious teeth and two sound teeth were not restored and used as a negative controls, one half of each tooth was stored in Tetracycline-containing (T+) media, while the other half was stored in Tetracycline-free (T−) solution. All samples were stored separately in glass vials containing 7.0 ml of the storage media.

A two-photon fluorescence microscope was used to image the samples using a ×10/0.25 NA objective lens, 800 nm excitation wavelength, and 500 ± 20 nm emission filter. Using both fluorescence and fluorescence life-time imaging (FLIM), the dentin–cement interface of each sample was imaged at six points before aging and the *XY* coordinates of each point were saved. After 8 weeks of aging, samples were lightly polished with a 2400-grit carborundum paper and cleaned in an ultrasonic bath for 10 min to remove any precipitates that may have formed on the section surface; the same points were re-imaged after re-locating the sample at the previous position using the fiducial marks.

Fluorescence intensity (FI) was calculated for each image using Image J analysis software (ImageJ, Wayne Rasband, NIH, USA). The fluorescence intensity (FI) of the interfacial dentin underneath the restoration was measured and normalized to the FI measured for the dentin away from the interface. The ratio between the intensities in these two areas was calculated for each image and averaged for each sample. The average percentage of the change in FI before and after aging was calculated for each group. The change in FI was calculated using the following equation:

$$\frac{X_{a}-X_{b}}{X_{b}}\times 100\%$$

where *X*_*b*_ is the value before aging and *X*_*a*_ is the value after aging.

For the fluorescence lifetime (FLT) measurements, data were analyzed using TRI2 software (courtesy of Paul Barber, Grey Cancer Institute, Oxford). The decay curves were well fitted using a double exponential model. The analysis was conducted in the same manner as for the FI, where an area of interfacial dentin was selected while another area was selected away from the interface to represent the sound dentin. The change in the FLT after aging was calculated using the above equation, and values were averaged for each sample and each group.

**Fig. 4:**
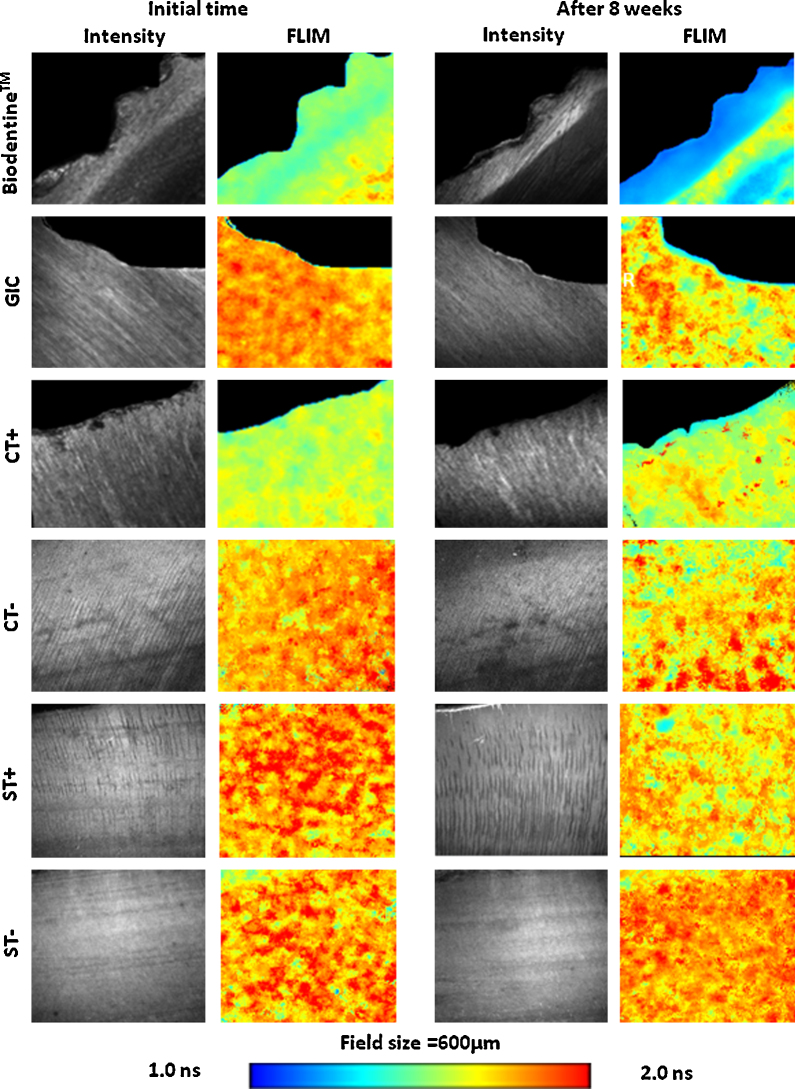
Representative fluorescence intensity (FI) and fluorescence lifetime (FLT) images for the dentin–cement interface of different groups before and after aging. A well defined band of shorter fluorescence life-time was detected in the (Biodentine™) filled samples. Reduction of the fluorescence lifetime was also detected in GIC filled samples as a band below the interface with more areas infiltrating the dentin matrix. The restoration-free samples (CT+, ST+) that were stored in Tetracycline containing solution, showed minimal and more generalized fluorescence changes. Other controls stored in Tetracycline free solution (CT−, ST−) showed non-significant changes in FLT (R = restoration, C = excavated caries, S = sound, T = Tetracycline).

Representative fluorescence intensity and lifetime images of each group before and after aging are shown in Fig. 4. The blue color in the FLIM images represents the drop in the FLT. The reduction in the FLT of the dentin could be explained by the adsorption or incorporation of Tetracycline, which has a very short FLT compared with the FLT of dentin. However, in the Biodentine™ group the area of reduced FLT appeared in the form of a well-defined band underneath the dentin–cement interface, which also appeared in the GIC filled samples. On the contrary, in the samples without restoration (CT and ST), the reduction in the FLT was generalized. The appearance of the bands indicates that Tetracycline incorporation was mainly concentrated in these areas, which could be explained by active mineral deposition and remineralization of the matrix.

**Fig. 5:**
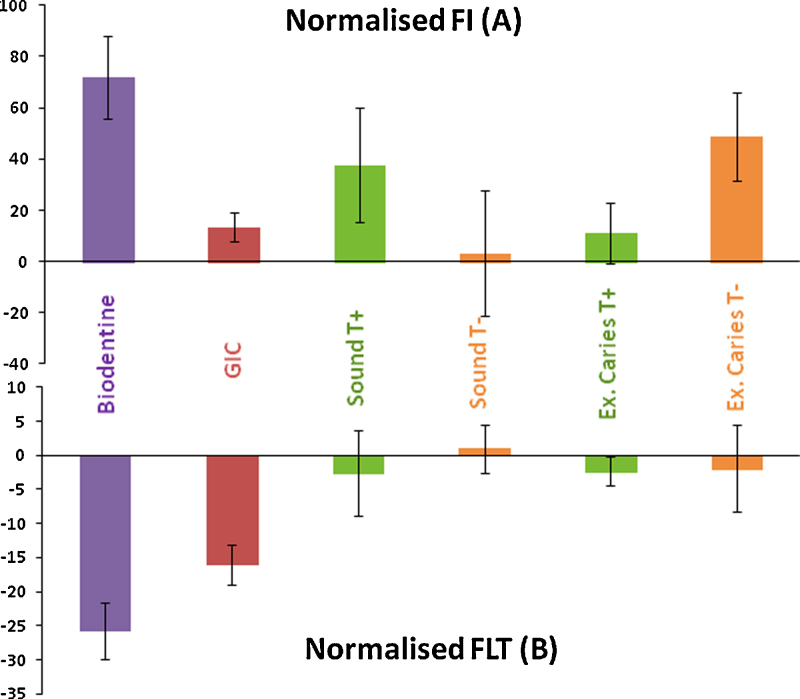
Normalized fluorescence intensity (FI) and fluorescence lifetime (FLT) presented as percentage differences of samples restored with Biodentine™, GIC, sound teeth – with and without Tetracycline and excavated carious teeth – with and without Tetracycline.

The effect of the aging of caries-affected dentin or sound dentin on the FI and FLT when stored in phosphate-rich media with or without the Biodentine™ or GIC is presented in Fig. 5. The graph in Fig. 5a shows an increase in the FI in all of the samples. Biodentine™-filled samples exhibited the highest increase (71%), while in the GIC samples the increase was around (13%). The FI increase in the restoration-free carious samples stored in Tetracycline-free media (CT−) was found to be higher (48.5%), while it changed minimally in the sound samples stored in the same media (ST−) or in the carious sample stored with Tetracycline.

The FLT graph in Fig. 5b shows a reduction in the normalized FLT in all of the groups except for the restoration-free sound dentin stored Tetracycline-free (T−) PBS solution. The reduction in the Biodentine-filled samples was the highest (−25.5%), followed by the GIC-filled samples (−16%) compared to the minimal changes in the control samples (−2%).

In this study, all of the teeth that were used were selected with deep carious lesions. However, since these lesions occurred naturally, it was impossible to fully standardize caries excavation, which may have led to a variation in the quality of the tissues left at the end of the caries excavation. Under or over excavation of carious dentin could have left caries-infected or sound dentin respectively. If infected dentin was left, fluorophores originating from the cariogenic bacteria or from the host might interfere with the FLIM analysis. Moreover, residual infected dentin could interfere with the remineralization process, where no remineralization is expected to occur in this structure-less substrate. On the other hand, over-excavation would remove the caries affected dentin and leave a sound substrate, with no potential sites for remineralization. Normalization of the FI and FLT measurements of the interfacial dentin to similar measurements obtained from areas of sound tissue away from the interface made the results more comparable, where the sound dentin could be considered as an internal control for the same sample, and samples of the same group.

Increased fluorescence intensity and shortened fluorescence lifetime of the interfacial dentin are both related to the incorporation of Tetracycline fluorophore into the dentin at these areas. Such incorporation could be mediated by the formation of calcium (or strontium in case of the GIC) containing minerals such as hydroxyapatite, which is expected to form in the presence of calcium and phosphate under high pH conditions. Such conditions were available in the Biodentine™ group, in which the hydrated cement was the source of calcium and hydroxyl ions, and the phosphate buffered saline was the source of the phosphate ions. This explains the change in the FLT in this group. While in the absence of free calcium ions, as in the restoration-free samples (CT+), there was almost no change in the FI and FLT before and after aging. In the caries-excavated samples stored in Tetracycline-free media (CT−), bacterial growth over or within the samples cannot be excluded with the absence of Tetracycline which could play this role in the aging media, therefore this growth might have resulted in an increase in the FI after storage, with minimal changes in the FLT. With the sound dentin samples, as expected there were no changes in the samples lacking Tetracycline (ST−). However, a higher increase in FI with (ST+) samples were found and could be attributed to the Tetracycline conditioning effect on sound dentin. This role of Tetracycline was observed in periodontal studies using Tetracycline for dentin conditioning to facilitate dental tissue healing [72], [73].

In the Biodentine™ and GIC samples, the increase in the FI of interfacial dentin after aging indicated the formation of Tetracycline-incorporated minerals, which could indicate remineralization. The difference in the FI between the two groups could be attributed to the nature of the minerals that has formed, as the pH conditions were not the same between the two groups. In the Biodentine™ filled samples, the storage solution developed a high pH that favors the formation of hydroxyapatite. In the GIC filled samples (GT), however, the pH was much lower, which would be less favorable for apatite formation. This could also explain the difference in the FLT change between the two groups.

This study indicates that fluorescence lifetime and fluorescence intensity imaging and measurements can produce a useful insight into mineralization processes, recorded over time, within the same carious lesion next to bioactive restorative materials. The future challenge will be to develop imaging and measurement techniques that can assess the interface of the whole carious lesion, both infected and affected dentin, with materials that are capable of encouraging repair and replacement of lost tooth structure.

## 8 Conclusions

This paper has reviewed the underlying chemistry and the interactions between glass ionomer cements and calcium silicate cements with tooth tissue, concentrating on the dentin–restoration interface. The local bioactivity of these materials can produce mineralization within the underlying dentin substrate. The advantage of this for the minimally invasive management of carious dentin is self-evident. We also report a new high resolution imaging technique that can be used to show mineralization within dental tissues, while also allowing monitoring of samples over time. There is a clear need to improve the bioactivity of our restorative dental materials and these cements offer exciting possibilities in realizing this goal.

## Conflict of interest

The author(s) declare no potential conflicts of interest with respect to the authorship and/or publication of this article.

## Ethics approval

Ethics approval was granted: reference number 12/LO/0290 NRES Committee London (Stanmore) for the use of extracted teeth.
